# Factors influencing hygienic behavior among students in Central Kazakhstan during COVID-19

**DOI:** 10.3389/fpubh.2025.1593579

**Published:** 2025-08-29

**Authors:** Zhanerke Bolatova, Marat Kalishev, Karina Nukeshtayeva, Olzhas Zhamantayev, Zhaniya Dauletkaliyeva, Gaukhar Kayupova, Nurbek Yerdessov, Hans Orru

**Affiliations:** ^1^School of Public Health, Karaganda Medical University, Karaganda, Kazakhstan; ^2^Institute of Family Medicine and Public Health, University of Tartu, Tartu, Estonia

**Keywords:** handwashing, hygiene, students, COVID-19, behavior

## Abstract

**Introduction:**

Hand hygiene is a fundamental public health measure that plays a crucial role in preventing the spread of infectious diseases, particularly in communal settings such as schools. The COVID-19 pandemic heightened awareness of hygiene practices, emphasizing the need for sustained interventions to ensure long-term adherence and improved health outcomes among students. This research aims to examine the key factors that shaped students’ hygiene behaviors during the COVID-19 pandemic and evaluate strategies for reinforcing and sustaining these practices over time.

**Methods:**

This study utilized a cross-sectional design to evaluate hand hygiene practices and influencing factors among 3,980 students from grades 5, 9, and 11 in the Karaganda region of Kazakhstan during the COVID-19 pandemic. Data were analyzed using IBM SPSS Statistics 26.0, with Chi-square tests assessing group differences and binary logistic regression identifying factors associated with students’ handwashing behaviors.

**Results:**

The results revealed that several factors, including school discussions on handwashing, school type (urban vs. rural), gender, grade level, and parental occupation and education, were significantly associated with improved handwashing behavior among students. Urban students, females, higher-grade students, and those with highly educated or employed parents demonstrated better hand hygiene practices, with school-based discussions on handwashing increasing the odds by 38%.

**Conclusion:**

This study underscores the critical role of educational interventions, sociodemographic factors, and infrastructure in shaping students’ hand hygiene behaviors, emphasizing the need for sustained efforts to reinforce proper handwashing practices through school-based education, policy support, and improved hygiene facilities.

## 1 Introduction

Hygiene plays a critical role in public health, serving as a foundational element in the prevention of disease and the promotion of overall well-being. The World Health Organization (WHO) and the Centers for Disease Control and Prevention (CDC) emphasize that effective handwashing with soap and clean water is a key intervention in reducing disease transmission (1, 2). The COVID-19 pandemic significantly increased awareness and adherence to hand hygiene practices, emphasizing their importance not only in preventing COVID-19, but also in reducing the transmission of other infectious diseases (3, 4). Beyond COVID-19 prevention, improved handwashing practices have contributed to a decline in various infectious diseases such as gastrointestinal infections and respiratory infections, underscoring the long-term benefits of hygiene awareness (5).

Hand hygiene is particularly critical in school environments, where students interact closely, creating opportunities for disease transmission (6). Studies have shown that proper handwashing can reduce respiratory infections by up to 21% and gastrointestinal infections by 31% among schoolchildren (7, 8). Additionally, interventions promoting hand hygiene in schools have been effective in reducing *Escherichia coli* contamination and lowering incidences of diarrheal diseases and respiratory infections (9–11). Despite these benefits, many schools, especially in low- and middle-income countries, lack the necessary infrastructure, such as handwashing stations and consistent access to clean water, to support effective hygiene practices (12, 13). The COVID-19 pandemic further exacerbated these challenges, particularly in disadvantaged area, where schools struggled to maintain hygiene facilities and supplies (14).

Behavioral factors also play a crucial role in students’ adherence to hand hygiene. Studies suggest that children are more likely to wash their hands if they perceive it as a social norm among their caregivers, highlighting the impact of peer influence (15, 16). Additionally, structured hygiene curricula, strategically placed handwashing stations, and teacher-led demonstrations have been shown to significantly improve handwashing frequency among students (17, 18). However, despite increased awareness during the COVID-19 pandemic, sustaining improved hygiene behaviors remained challenging, especially among younger students, who may not fully grasp the long-term health benefits of handwashing (19, 20).

Although hygiene’s role in preventing infectious diseases is well-documented, there is a lack of comprehensive research evaluating school students’ hygiene behaviors in Central Asian countries, particularly during the COVID-19 pandemic. Existing studies have primarily focused on general public health measures, rather than the behavioral responses of students to pandemic-driven hygiene interventions. Moreover, little is known about the factors influencing students’ long-term adherence to hygiene practices once emergency health measures are removed. Kazakhstan, as one of the largest and most socioeconomically diverse countries in Central Asia, serves as a valuable case study for understanding these dynamics (21, 22). The findings of this study can contribute to a broader understanding of hygiene behaviors in the Karaganda region in Central Kazakhstan and inform the development of effective, long-term hygiene interventions in educational settings.

This research aims to identify the key factors that influenced students’ hygienic behavior during the COVID-19 pandemic and assess how these behaviors can be reinforced, and sustained over time. By providing evidence-based insights, the study will contribute to school-based public health interventions and broader hygiene promotion strategies, ensuring that the benefits of heightened hygiene awareness during the pandemic translate into lasting public health improvements.

## 2 Materials and methods

### 2.1 Study area

**Figure 1:**
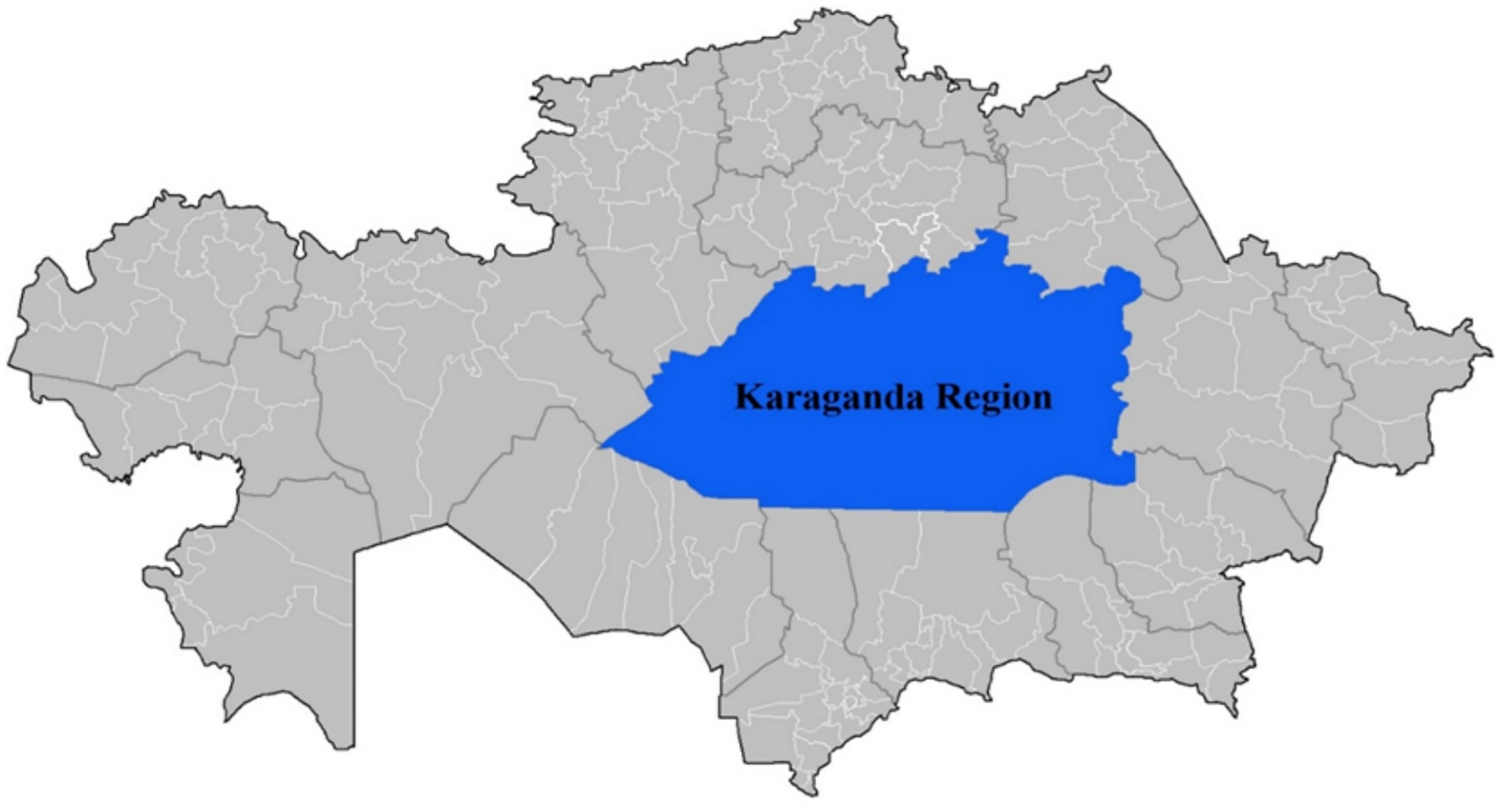
The location and borders of the Karaganda region (selected area) on Kazakhstan map (before the 8 June, 2022). NB - the study was conducted before the division of the region.

The Karaganda Region is one of the largest administrative divisions of Kazakhstan, situated in the center of the Eurasian continent (Figure 1). Geographically, the region is equidistant from the Arctic, Indian, Atlantic, and Pacific Oceans, which determines its sharply continental climate. The region covers the most elevated part of the Kazakh Hills—Saryarka (23).

The administrative center of the region is the city of Karaganda, founded in 1934. Today, the Karaganda region is one of the industrial regions of Kazakhstan, as it is rich in minerals and raw materials. It occupies 239,045 square km, which contains 8.7 of the whole territory of the Republic. As of January 1, 2025, the population of the region was 1,134.0 thousand people, of whom 929.8 thousand (82%) resided in urban areas, while 204.2 thousand (18%) lived in rural areas (24). 504 public schools operate in the Karaganda region. The number of students was about 204 thousand (204,174) in 2020 (25).

The region comprises eight cities, including six of regional significance and two of district significance. Additionally, there are eight settlements and 349 rural localities within the region.

The climate is sharply continental, characterized by 5.5 months of long cold winter and highly arid, with 3 months of hot summer. Temperature is about −16–17 °C in winter and 20–21 °C in summer. On summer days, the temperature can rise to 37 degrees. The coldest month is January. Frosts reach up to 40 degrees. Annual precipitation in the region’s north is 250–300 mm; in the south – 150–210 mm; in the low mountainous areas – 300–400 mm. It rains mainly from April to October.

### 2.2 Study population

This study employed a cross-sectional design to assess hand hygiene practices and associated factors among students in grades 5, 9, and 11 during the COVID-19 pandemic. The study population comprised 3,980 students enrolled in schools within the Karaganda region of Kazakhstan. Inclusion criteria specified students from grades 5, 9, and 11, attending either rural or urban schools within the region. A simple random sampling method was employed to select participants, ensuring an equal chance of inclusion for all eligible students. The overall response rate was 88%; however, 523 responses were excluded from the analysis due to incomplete data submission.

The study was conducted online among students attending schools in Karaganda region, Central Kazakhstan, between April 2 and May 20, 2021. An electronic questionnaire was disseminated to teachers through school administrations and subsequently completed by students. It was carried out during the resumption of face-to-face education in schools across Kazakhstan amid the COVID-19 pandemic (26). According to the resolution, in regions classified as high-risk zones, combined-format education was implemented. This included face-to-face instruction for preschool students and grades 1 to 5 with class sizes limited to 25 children, and a hybrid format for graduating classes (grades 9 and 11), with 70% of lessons conducted in person and 30% online. Strict sanitary, disinfection, and mask compliance measures were followed, with a limit of 15 students per class and adherence to the “1 class - 1 room” principle.

Prior to data collection, informed consent forms were distributed to parents through students in sealed envelopes to obtain written parental consent. The signed forms were returned to the teachers via the students. A collaboration plan was coordinated with school administrators to facilitate data collection. Participation in the study was voluntary, and students were informed about the study’s purpose before verbal assent was obtained. Data were collected online within the classroom environment, following a prearranged schedule. Completing the survey took approximately 10–15 min per participant.

### 2.3 Data analysis

The socio-demographic characteristics of the participants were assessed through seven questions, which addressed age, gender, grade level, and the educational and employment status of their parents. Hand Hygiene Behavior Form consisted of nine items designed based on existing literature (19, 27–29). The questions aimed to evaluate participants’ hand hygiene behaviors during the COVID-19 pandemic. The form was based on the WHO/UNICEF framework “Surveillance of Water, Sanitation and Hygiene in Schools,” with selected items from the hygiene section adapted for use in this study (27). The questions were modified to reflect the local school context and to ensure cultural appropriateness. Content validity was assessed through expert review by public health professionals and school administrators. The instrument was translated into both Kazakh and Russian, followed by back-translation to ensure linguistic accuracy and conceptual consistency. A pilot test was conducted with a small group of students to evaluate the clarity and comprehensibility of the items. Minor revisions were made based on the pilot feedback prior to full-scale implementation. The items focused on specific handwashing circumstances and procedures. Respondents were asked to indicate whether they washed their hands in particular situations, including, when hands were visibly dirty, before meals, after using the toilet, after playing with pets, after contact with an unwell individual, after using public transportation, upon returning home, and when washing hands with soap and water. Issues associated with inadequate handwashing were also explored. Responses were scored on a binary scale, with “Yes” assigned 1 point and “No” assigned 0 points, yielding a total possible score range of 0 to 9. The internal consistency reliability of the Hand Hygiene Behavior Form was determined to be 0.79, indicating acceptable reliability for this assessment tool.

Statistical analysis was performed using IBM SPSS Statistics 26.0 package program. Group differences were assessed using the Chi-square test (χ^2^) to evaluate statistical significance. A binary logistic regression was conducted to identify factors associated with hand hygiene practices. The dependent variable was students’ handwashing behavior, with independent variables comprising a range of demographic factors. The statistical significance level was taken as 0.05 in all tests. In order to obtain statistical results.

### 2.4 The ethical principles of the study

To conduct the study, ethical approval was obtained from the Bioethics Committee at Karaganda Medical University (approval dated 12/04/2021, No 18), with legal permission from the Department of Education of the Karaganda Region. The survey was administered in both Kazakh and Russian to accommodate the linguistic diversity of students in the region’s schools.

Following the collection of written parental consent, students were informed about the purpose and voluntary nature of the study. It was emphasized that participation was entirely optional, and students could withdraw from the study at any time without facing any consequences. Confidentiality of personal information was guaranteed, and students were assured that non-participation would not affect their grades or academic standing.

## 3 Results

### 3.1 Scenarios for assessing handwashing behavior

Nine survey items were used to assess handwashing practices among students (Table 1). The findings revealed that 85.3% of respondents consistently wash their hands, when visibly dirty and 90% reported regular handwashing with soap and water. The majority of participants demonstrated an understanding of the potential health risks associated with inadequate hand hygiene. Additionally, 60% of students reported washing their hands before meals, upon returning home, and after using the toilet. In contrast, 40% of students reported handwashing after interacting with pets or using public transportation. Only 24.4% of students practiced hand hygiene following contact with an unwell individual.

**Table 1:** Scenarios for assessing handwashing behavior.

Handwashing scenarios		Total, *N* = 3,980		Female, *n* = 2,439		Male, *n* = 1,541		Chi square; *p*-value
		*n*	%	*n*	%	*n*	%	
Always when hands are dirty	Yes	3,396	85.3	2,120	53.3	1,276	32.0	12.787; 0.0001
	No	584	14.7	319	8.0	265	6.7	
Before meals	Yes	2,308	58.0	1,488	37.4	820	20.6	23.562; 0.0001
	No	1,672	42.0	951	23.9	721	18.1	
After using the toilet	Yes	2,724	68.4	1748	43.9	976	24.5	30.362; 0.0001
	No	1,256	31.6	691	17.4	565	14.2	
After playing with pets	Yes	1,543	38.8	1,014	25.5	529	13.3	20.887; 0.0001
	No	2,437	61.2	1,425	35.8	1,012	25.4	
After contact with an unwell friend	Yes	971	24.4	624	15.7	347	13.3	4.814; 0.028
	No	3,009	75.6	1815	45.6	1,194	30.0	
After using public transport	Yes	1,586	39.8	1,060	26.6	526	25.5	34.271; 0.0001
	No	2,394	60.2	1,379	34.6	1,015	25.5	
Upon returning home	Yes	2,239	56.3	1,482	37.2	757	19.0	51.982; 0.0001
	No	1741	43.7	957	24.0	784	19.7	
Hand washing with soap and water	Yes	3,534	88.8	2,225	55.9	1,309	32.9	37.442; 0.0001
	Only with water	446	11.2	214	5.4	232	5.8	
Issues arising from not washing hands	Yes	3,840	96.5	2,391	60.0	1,449	36.4	44.568; 0.0001
	No	140	3.5	48	1.2	92	65.7	

A significant disparity in handwashing practices was identified between female and male students. Female students demonstrated a higher frequency of handwashing across various situations, with chi-square test results indicating statistically significant differences between the girls and boys (*p* ≤ 0.05). Handwashing behavior was classified as “adequate” when respondents affirmed at least seven of the assessed items. Conversely, responses with fewer than seven affirmative answers were categorized as “inadequate” handwashing behavior. This threshold is consistent with scoring approaches used in prior cross-sectional studies on student hygiene behavior during COVID-19, where engaging in at least two-thirds to three-quarters of recommended practices was treated as sufficient for classification as “adequate” hygiene [19, 28, 29].

Overall, 40.1% of students demonstrated adequate hand hygiene practices (*n* = 1,597). Results from the chi-square analysis indicated statistically significant associations between hand hygiene adequacy and variables, such as school location (*χ*^2^ = 81.476, *p* < 0.01), gender (*χ*^2^ = 39.225, *p* < 0.01), grade level (*χ*^2^ = 23.335, *p* < 0.01), father’s occupation (*χ*^2^ = 28.007, *p* < 0.01), mother’s occupation (*χ*^2^ = 37.903, *p* < 0.01), mother’s educational level (*χ*^2^ = 45.915, *p* < 0.01), father’s educational level (*χ*^2^ = 43.523, *p* < 0.01), and discussions on handwashing at school (*χ*^2^ = 17.448, *p* < 0.01).

### 3.2 Demographic characteristics and comparative analysis of hand hygiene practices among school children

The analysis included data from 3,980 students, with a 63.9% being female (*n* = 2,439) and 36% (*n* = 1,375) from urban schools. The distribution across grade levels showed that half of the respondents were from the 9th grade (50.5%, *n* = 2007) (Table 2).

**Table 2:** Comparative analysis of the adequacy of hand hygiene practices.

Category	Group	Adequate rate of hand hygiene		Inadequate rate of hand hygiene		Chi square; *p*-value
		*n*	%	*n*	%	
Educational institutions	Rural	419	10.5	956	24.0	81.476; 0.0001
	Urban	1,178	29.6	1,427	35.6	
Year of study	5	261	6.6	506	12.7	23.335; 0.0001
	9	795	20.0	1,212	30.5	
	11	541	13.6	665	16.7	
Gender	Male	524	13.2	1,017	25.6	39.225; 0.0001
	Female	1,073	30.0	1,366	34.3	
Mother’s employment	Unemployed/Temporarily unemployed	416	10.5	683	17.2	37.903; 0.0001
	Professional and technical personnel	394	10.0	574	14.4	
	Civil servant	169	4.3	323	8.1	
	Company employee	214	5.4	248	6.2	
	Head/Manager of governmental bodies and institutions	20	0.5	23	0.6	
	Entrepreneur	128	3.2	126	3.2	
	Service sector employee	232	5.8	334	8.4	
	Different occupations	24	0.6	72	1.8	
Father’s employment	Unemployed/Temporarily unemployed	125	3.1	263	6.6	28.007; 0.0001
	Professional and technical personnel	258	6.5	379	9.5	
	Civil servant	234	5.9	376	9.5	
	Company employee	391	9.8	483	12.1	
	Head/Manager of governmental bodies and institutions	35	0.9	30	0.8	
	Entrepreneur	210	5.3	279	7.0	
	Service sector employee	183	4.6	308	7.7	
	Different occupations	161	4.0	265	6.7	
Mother’s educational background	Secondary level education	455	11.4	894	22.5	45.915; 0.0001
	Specialized secondary education	288	7.2	389	9.8	
	Incomplete higher education	56	1.4	95	2.4	
	Higher education	775	19.5	949	23.8	
	Master’s and doctoral studies	23	0.6	56	1.4	
Father’s educational background	Secondary level education	549	13.8	1,049	26.4	43.523; 0.0001
	Specialized secondary education	360	9.0	439	11.0	
	Incomplete higher education	57	1.4	105	2.6	
	Higher education	601	15.1	744	18.7	
	Master’s and doctoral studies	30	0.8	46	1.2	
Discussing the importance of handwashing	Yes	1,218	30.6	1,674	42.0	17.448; 0.0001
	No	379	9.5	709	17.8	

Regarding parental occupation, 27.7% of students’ mothers were unemployed, including housewives, while 24.4% were professional and technical personnel. Fathers showed higher occupational diversity: 21.9% were company employees, 16% were professional and technical personnel, and 12.3% each were businessmen and service sector employees.

In terms of education, 43.3% of mothers and 33.8% of fathers have higher education, with fathers more likely to have lower or upper secondary education (40.2%) compared to mothers (33.9%). Postgraduate education was rare, with only 2% of mothers and 1.9% of fathers attaining this level.

### 3.3 Factors associated with hand hygiene

In the binary logistic regression analysis, several factors, including handwashing discussions at school, school type (urban or rural), gender, grade level, and parental occupation and education showed significant associations with improved handwashing behavior (Table 3).

**Table 3:** Binary logistic regression analysis of factors influencing hand hygiene compliance (*n* = 3,980).

Variables		OR	OR 95% CI		*p*-value
			Lower	Upper	
Educational institutions	Urban	1.70	1.47	1.97	0.0001
Year of study	5				0.0001
	9	1.25	1.04	1.49	0.016
	11	1.53	1.26	1.86	0.0001
Gender	Female	1.52	1.33	1.75	0.0001
Mother’s employment	Unemployed/Temporarily unemployed				0.012
	Professional and technical staff	1.02	0.84	1.23	0.873
	Civil servant	0.84	0.66	1.06	0.147
	Company employee	1.12	0.89	1.42	0.337
	Head/Manager of governmental bodies and institutions	1.05	0.56	1.97	0.889
	Businesswoman	1.49	1.11	1.99	0.008
	Service sector employee	1.09	0.89	1.36	0.398
	Different occupations	0.59	0.36	0.97	0.039
Father’s employment	Unemployed/Temporarily unemployed				0.149
	Professional and technical staff	1.13	0.86	1.5	0.386
	Civil servant	1.29	0.97	1.70	0.077
	Company employee	1.38	1.06	1.79	0.017
	Head/Manager of governmental bodies and institutions	1.80	1.04	3.13	0.036
	Businessman	1.22	0.90	1.63	0.195
	Service sector employee	1.09	0.82	1.46	0.557
	Different occupations	1.24	0.91	1.67	0.169
Mother’s educational background	Secondary level education				0.003
	Specialized secondary education	1.19	0.96	1.48	0.12
	Incomplete higher education	1.13	0.78	1.65	0.52
	Higher education	1.39	1.15	1.67	0.001
	Master’s and doctoral studies	0.69	0.39	1.24	0.22
Father’s educational background	Secondary level education				0.116
	Specialized secondary education	1.23	1.01	1.50	0.042
	Incomplete higher education	0.92	0.64	1.32	0.636
	Higher education	1.19	0.99	1.44	0.054
	Master’s and doctoral studies	1.39	0.81	2.42	0.231
Discussing the importance of handwashing	yes	1.38	1.19	1.60	0.0001
VIF < 3					

Urban school students demonstrated 1.70 times (95% CI = 1.47–1.97, *p* < 0.01) better handwashing behavior than those in rural schools. Gender differences were also evident, with female students being 1.52 times (95% CI = 1.33–1.75, *p* < 0.01) more likely to practice better handwashing compared to male students.

Grade level had a notable impact, as students in grade 9 were 1.25 times (95% CI = 1.04–1.49, *p* < 0.05) and those in grade 11 were 1.53 times (95% CI = 1.26–1.86, *p* < 0.01) more likely to exhibit good handwashing habits compared to students in grade 5.

Parental occupation also played a significant role. Students, whose mothers were businesswomen demonstrated 1.49 times (95% CI = 1.11–1.99, *p* < 0.01) better handwashing behavior than those with temporarily unemployed mothers. In contrast, students whose mothers selected “other” for occupation were 41% less likely to exhibit good handwashing behavior (OR = 0.59, 95% CI = 0.36–0.97, *p* < 0.05) compared to those whose mothers were unemployed.

Similarly, students whose fathers were company employees or held managerial positions in governmental bodies were more likely to have better handwashing behavior, with ORs of 1.38 (95% CI = 1.06–1.79, *p* < 0.05) and 1.80 (95% CI = 1.04–3.13, *p* < 0.05), respectively, compared to students with temporarily unemployed fathers.

Parental education was another determinant, with students whose mothers had a high level of education 1.39 times (95% CI = 1.15–1.67, *p* < 0.01) more likely to report good handwashing habits than those whose mothers had only secondary education. Fathers with secondary special education (OR = 1.23, 95% CI = 1.01–1.50, *p* < 0.05) and higher education (OR = 1.19, 95% CI = 0.99–1.44, *p* < 0.05) were also associated with better handwashing behavior among students, compared to those with secondary education.

The OR for students, who reported discussions about the importance of handwashing at school was 1.38 (95% CI = 1.19–1.60, *p* < 0.01), indicating a 38% higher odds of better handwashing behavior compared to students without such discussions.

## 4 Discussion

This study provides valuable insights into hand hygiene practices among school students in Kazakhstan, highlighting key demographic, behavioral, and educational factors that influence compliance.

The majority of students demonstrated appropriate hand hygiene practices, consistently washing their hands when visibly dirty and using soap and water. This high compliance aligns with public health guidelines, as proper handwashing is one of the most effective measures for reducing the spread of infectious diseases, including respiratory and gastrointestinal infections (30–32). Additionally, nearly all students exhibited awareness of the health risks associated with inadequate hand hygiene, reinforcing the link between knowledge and hygiene behaviors. Prior research suggests that awareness of disease transmission pathways and the protective role of handwashing significantly contributes to sustained behavioral improvements (33–35). Despite this awareness, gaps in routine hygiene behaviors persist. Only 60% of students practiced handwashing before meals and after returning home, and just three out of five students washed their hands after playing with pets or using public transportation. These findings highlight inconsistencies in compliance, particularly in contexts where students may not perceive an immediate health risk. The Health Belief Model (HBM) provides a useful framework for interpreting this behavior, as it suggests that individuals are more likely to adopt preventive measures when they perceive a high susceptibility to illness and believe that the benefits outweigh the barriers (36).

A particularly concerning is that only 25% of students in Karaganda region practiced hand hygiene after contact with a sick friend, posing a significant risk for disease transmission, particularly in school environments where close interactions facilitate the spread of infections (37–39). This discrepancy between knowledge and practice suggests that interventions should focus not only on education, but also on behavioral reinforcement strategies, such as habit formation techniques and peer modeling, to enhance adherence to critical hygiene practices. The study also identified notable gender differences, with female students exhibiting significantly better hand hygiene practices than males. This finding is consistent with global research suggesting that girls tend to engage in hygiene behaviors more diligently than boys (40–42). Several factors may contribute to this trend, including social norms, parental reinforcement, and gendered health perceptions, which often encourage hygiene-conscious behaviors more strongly among girls than boys.

It appeared that urban students demonstrated better hand hygiene practices than their rural counterparts. A similar trend has also observed in previous studies from Ethiopia and other regions (43). This disparity may be attributed to differences in access to water, sanitation infrastructure, and hygiene education programs. Schools in urban settings in Kazakhstan typically benefit from better hygiene facilities, structured health curricula, and stronger public health initiatives, whereas rural schools often face resource constraints.

The findings of this study indicate that handwashing behavior improves with increasing grade level, as older students demonstrated greater adherence to proper hygiene practices compared to younger students (28, 44). Prior research has also established a positive correlation between educational level and hygiene compliance, indicating that students in higher grades are more likely to internalize and consistently practice proper handwashing habits (45).

Parental education emerged as a key determinant of students’ hand hygiene behaviors. Students with more highly educated parents—particularly mothers—demonstrated significantly better hygiene compliance, aligning with existing research suggesting that higher parental educational attainment correlates with improved health behaviors among children (46, 47). Educated parents are more likely to emphasize hygiene at home, provide access to proper sanitation resources, and model good handwashing behavior of children. Additionally, maternal occupation was linked to students’ hand hygiene habits, potentially reflecting differences in health literacy, exposure to hygiene-related information, and household health priorities across occupational groups (48).

Students who engaged in discussions about handwashing at school demonstrated higher compliance, emphasizing the importance of education and awareness in shaping hygiene behaviors. Research consistently highlights that structured hygiene education—whether delivered through school curricula, peer discussions, or teacher-led initiatives—significantly improves adherence to hand hygiene practices (49–52). Integrating hand hygiene education into school programs and ensuring that teachers actively reinforce these lessons can help sustain long-term behavioral change. Moreover, behavioral interventions such as habit formation strategies, nudges (e.g., reminder posters near sinks), and positive reinforcement techniques can help bridge the gap between knowledge and practice. Empirical evidence suggests that motivational strategies, combined with accessible infrastructure (readily available soap and water), enhance compliance and long-term sustainability of hygiene behaviors (53).

The integration of behavioral theories provides a valuable lens for interpreting the determinants of handwashing behavior among school-aged children. The Theory of Planned Behavior (TPB) posits that behavioral intention is shaped by attitudes, subjective norms, and perceived behavioral control—all of which are relevant in the school context (54, 55). Positive attitudes toward hand hygiene, often fostered through health education, have been associated with greater compliance (48, 56). Similarly, subjective norms—reinforced through peer influence and support from educators—play a pivotal role in shaping normative beliefs and behavior (57, 58). Perceived behavioral control, including the availability of handwashing facilities and opportunities for skill reinforcement, enhances self-efficacy and behavioral consistency (8, 59). Complementing TPB, Social Cognitive Theory (SCT) emphasizes self-efficacy, observational learning, and social influences as core constructs influencing behavior. Evidence suggests that modeling, peer-led demonstrations, and environmental cues can significantly enhance hand hygiene practices (11, 16, 60). Together, TPB and SCT underscore the importance of multifaceted, theory-driven interventions that combine individual motivation, peer dynamics, and structural facilitators to support sustained handwashing behavior in school settings.

At a policy level, implementing comprehensive WASH programs in schools, particularly in underserved areas in Kazakhstan, is essential for improving public health outcomes (61, 62). Investments in hygiene infrastructure, teacher training, and community-based awareness campaigns can help ensure that hygiene promotion becomes a sustained public health priority rather than a reactive response to health crises. This study makes a significant contribution to public health research by examining hand hygiene behaviors among school students during the COVID-19 pandemic, a period of heightened awareness but also considerable logistical challenges in data collection. Despite these challenges, the study successfully gathered data from nearly 4,000 students, offering a large and representative dataset that provides valuable insights into hygiene behaviors in a high-risk context in Central Asia.

However, certain limitations and targeted recommendations for future research must be acknowledged. A notable aspect is the higher proportion of female students in the sample, which does not align with the overall gender distribution of children in Kazakhstan, where male children slightly outnumber female children (24). Additionally, urban students were overrepresented compared to their rural counterparts, a trend that reflects Kazakhstan’s population distribution. The overrepresentation of female students and urban schools in the sample may have biased the results, particularly if hygienic behaviors or access to hygiene facilities differ systematically by location. Female students may be more likely to report or engage in certain health-related behaviors, and urban schools may have better hygiene infrastructure and awareness programs compared to rural counterparts. As a result, the findings may overestimate the overall level of hygienic behavior among schoolchildren in Kazakhstan. Future research should aim to address these disparities by employing stratified sampling strategy to ensure representative inclusion of male and rural students. Moreover, future research should consider employing multilevel or hierarchical modeling to account for potential school-level clustering, which may influence student behavior and associated predictors. The reliance on self-reported data introduces the potential for social desirability bias and recall bias, as students may have over-reported their adherence to hygiene practices due to perceived social expectations. Although measures were taken to mitigate this bias—such as ensuring respondent anonymity and emphasizing the confidentiality of responses—some inflation of reported compliance is likely. Future research should consider the use of objective assessment methods, including direct observation or sensor-based monitoring, to enhance the validity and reliability of behavioral data. Additionally, as a cross-sectional study, the findings capture hygiene behaviors at a single point in time, limiting the ability to assess long-term behavioral changes. Specifically, while associations with variables such as parental education or school location may suggest influences on behavior, we cannot determine the directionality or permanence of these effects. As such, interpretations regarding sustained behavior change over time should be made with caution. Future research should consider longitudinal studies to examine how hand hygiene behaviors evolve over time and identify socio-cultural factors that influence sustained compliance. Additionally, qualitative research could provide deeper insight into the motivations, contextual influences, and barriers affecting student hygiene practices. Although no formal knowledge translation initiatives were implemented, preliminary findings were informally shared with participating schools and discussed with local education staff, and a WASH course was developed and distributed in select institutions. While these efforts contributed to initial engagement, the lack of a structured dissemination strategy limits broader impact. Future studies should prioritize systematic knowledge translation approaches to support policy translation and sustainable school-based interventions.

## 5 Conclusion

Current study highlights positive trends and areas for improvement in the hand hygiene behaviors of school students in Kazakhstan. While self-reported awareness of handwashing is high, significant gaps remain in routine practices. The study identifies key factors influencing students’ handwashing behavior, highlighting the role of sociodemographic and educational determinants. Students from urban schools, female students, those in higher grade levels, and those with parents who are employed and have higher education levels demonstrated significantly better handwashing practices. Furthermore, school-based discussions on handwashing were strongly associated with improved hygiene behavior, underscoring the importance of educational interventions in promoting proper hand hygiene among students.

Strengthening hand hygiene requires a combination of education, behavioral reinforcement, and infrastructure improvements. Schools should integrate structured hygiene education, peer-led discussions, and teacher reinforcement, while community-based awareness programs can further support hygiene promotion. Investments in WASH infrastructure, particularly in underserved areas, and longitudinal research on sustainable behavior change are essential for long-term impact.

The COVID-19 pandemic reinforced the importance of hand hygiene in preventing disease transmission, underscoring the need for sustained interventions beyond the crisis. Handwashing must be ingrained as a lifelong habit through education, policy support, and behavioral reinforcement. Schools, policymakers, and health authorities must collaborate to ensure access to hygiene facilities and structured education programs. Investing in hygiene education and infrastructure not only reduces infection risks but also fosters a healthier, more resilient generation. The lessons learned must drive permanent improvements in hygiene practices, ensuring that clean hands save lives every day, not just in times of crisis.

## Data Availability

The original contributions presented in the study are included in the article/Supplementary material, further inquiries can be directed to the corresponding authors.
