# Supplementary material for: Factors influencing hygienic behavior among students in Central Kazakhstan during COVID-19
Source: Front Public Health. 2025 Aug 29;13:1593579. doi: 10.3389/fpubh.2025.1593579 (PMC12426162; doi:10.3389/fpubh.2025.1593579)
Supplement: Supplementary file 1 [file Data_Sheet_1.pdf]

Supplementary Material

Investigation on Handwashing Behavior of School Students

Dear Participant,
You are invited to take part in an anonymous survey conducted as part of a research study aimed at assessing hand hygiene practices and related conditions among school students.
Participation in this study is entirely voluntary. The survey involves no risk to your health or academic performance. All responses will be treated with strict confidentiality and used solely for academic research purposes. The questionnaire consists of 12 items and will take only a few minutes to complete. We kindly ask that you respond based on your actual experiences and practices.
Your contribution is highly valued and will support the development of effective, evidence-based hygiene promotion strategies for school settings.
Thank you for your time and participation!

1.Would you like to participate in this survey?
□ Yes
□ No
2. Type of educational institution:
□ Rural
□ Urban
3. Please indicate your gender:
□ Female
□ Male
4. What grade are you in? _____
5. Mother's occupation:
□ Unemployed / temporarily not working
□ Professional/technical specialist (e.g., doctor, teacher, lawyer, engineer)
□ Government employee
□ Company employee
□ Executive/manager in public institutions or government bodies
□ Entrepreneur
□ Service sector employee
□ Other
6. Father's occupation:
□ Unemployed / temporarily not working
□ Professional/technical specialist (e.g., doctor, teacher, lawyer, engineer)
□ Government employee
□ Company employee
□ Executive/manager in public institutions or government bodies
□ Entrepreneur
□ Service sector employee
□ Other
7. Mother's education:
□ Secondary education
□ Specialized secondary education
□ Incomplete higher education
□ Higher education (university degree)
□ Postgraduate education

8. Father's education:
□ Secondary education
□ Specialized secondary education
□ Incomplete higher education
□ Higher education (university degree)
□ Postgraduate education
9. In which of the following scenarios did you wash your hands?
9.1. Whenever my hands are dirty
□ Yes
□ No
9.2. Before eating
□ Yes
□ No
9.3. After using the toilet
□ Yes
□ No
9.4. After playing with a pet
□ Yes
□ No
9.5. After contact with a friend who is feeling unwell
□ Yes
□ No
9.6. After using public transportation
□ Yes
□ No
9.7. After returning home from outside
□ Yes
□ No
10. If both water and soap are available, how do you usually wash your hands?
□ With water only
□ With water and soap
11**.** Have you ever been talked to at school about the importance of handwashing?
□ Yes
□ No
12. Do you know what problems can occur if you do not wash your hands?
□ Yes
□ No
